# Density functional theory in materials science

**DOI:** 10.1002/wcms.1125

**Published:** 2013-01-08

**Authors:** Jörg Neugebauer, Tilmann Hickel

**Affiliations:** * Correspondence to: neugebauer@mpie.de; Max-Planck-Institut für Eisenforschung GmbH, Department of Computational Materials Design, Max-Planck-Str. 1, 40237 Düsseldorf, Germany

## Abstract

Materials science is a highly interdisciplinary field. It is devoted to the understanding of the relationship between (a) fundamental physical and chemical properties governing processes at the atomistic scale with (b) typically macroscopic properties required of materials in engineering applications. For many materials, this relationship is not only determined by chemical composition, but strongly governed by microstructure. The latter is a consequence of carefully selected process conditions (e.g., mechanical forming and annealing in metallurgy or epitaxial growth in semiconductor technology). A key task of computational materials science is to unravel the often hidden composition–structure–property relationships using computational techniques. The present paper does not aim to give a complete review of all aspects of materials science. Rather, we will present the key concepts underlying the computation of selected material properties and discuss the major classes of materials to which they are applied. Specifically, our focus will be on methods used to describe single or polycrystalline bulk materials of semiconductor, metal or ceramic form.

## MATERIALS CLASSES

Within the focus of materials science, materials can be classified by various criteria. Depending on the type of chemical bonding and electronic structure, several major classes of materials are distinguished: metals (with no bandgaps) and semiconductors, ceramics, and polymers (with finite bandgaps). The border between semiconductors and ceramics is not well defined and their actual assignment is often determined by their specific application (e.g., in electronic and optoelectronic applications, a material may be considered a semiconductor, but in mechanical applications, a ceramic).

An alternative way to classify materials is with respect to whether their local atomic arrangement is highly ordered or not, and the size/regularity of their microstructure. Typically, one distinguishes perfect bulk crystals (e.g., Si-based microelectronics), polycrystals consisting of multiple single crystalline grains with sizes ranging from about 10 nm to several hundred μm, amorphous materials (e.g., glasses), soft matter (e.g., polymers), composites (e.g., biomaterials), and nanostructures. In the present paper, the focus will be on crystalline and polycrystalline materials on which density functional theory (DFT) has had a particularly strong impact.

Further to the above classification schemes, materials are also distinguished as functional and structural materials. Functional materials are highly sensitive in one or several of their properties to changes in the environment, whereas structural materials are optimized to withstand external forces. Table 1 classifies important groups of functional materials together with common simulation challenges.

**Table 1:** Classification of Selected Functional Materials with Respect to the Mechanism They Are Based on, Key Applications and Typical Questions Addressed by Density Functional Theory

Category	Applications	Simulation Challenges
Electronic	Microelectronics	Doping, defects, interfaces
Optical	Laser diodes, light emitting diodes	Bandstructure, matrix elements
Magnetic	Storage applications, spintronics, magnetocalorics	Magnetic structure, anisotropies, disorder
Mechanical	Structural components, shape memory effect, piezo- and pyroelectrics	Extended defects, elastic constants, complex energy landscapes, plasticity, disorder
Combinations	Multiferroics	

Although some of the challenges are unique to a specific material, a number of generic topics applying to several or all materials are apparent. They are not restricted just to functional materials, but apply equally well to structural materials. Examples of generic topics and structural motives are chemically ordered and disordered bulk materials, point defects (native defects, impurities/dopants), line defects (dislocations), planar defects (internal or external surfaces, homo- and heterointerfaces, grain boundaries, stacking faults), or quasi-zero-dimensional defects (precipitates, quantum dots). The behavior and the impact of such motives on materials behavior can be hugely different—for example, dislocations in semiconductors are highly detrimental to device performance whereas in many metallic alloys they are decisive for achieving high plasticity/ductility. Still the electronic structure/atomic scale methods, which have been developed to compute energetic stability, equilibrium structure, or mechanical or electronic properties, are often very similar. Although these properties have been historically addressed using *T* = 0 K formalisms, recent developments in efficient computation of accurate free energies allow extensions to finite temperatures.

## ELECTRONIC STRUCTURE

A key quantity of interest is the ground state (*T* = 0 K) total energy 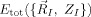 with the coordinates 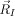 describing the atomic positions and *Z_I_* the atomic numbers (i.e., the chemical species). This quantity is directly accessible by most electronic structure approaches. For extended systems with spatial periodicity relevant for crystalline systems, DFT is the method of choice. One reason for this is that DFT relies solely on single-particle wavefunctions, which makes the implementation of periodicity straightforward. Second, modern implementations of DFT using plane waves together with pseudopotentials have for characteristic system sizes (i.e., a few hundred atoms) an effective scaling of O(*N^2^…N^3^*) with *N* the number of atoms.^1^ ^2^ ^3^ ^4^ For very large systems consisting of >10^3^ atoms, orthogonalization of the one-particle wavefunctions, which scales like O(*N*^3^), dominates the computation time. For such large system sizes, linearly scaling O(*N*) methods including tight-binding approaches have been developed.^5^,^6^

Although DFT has been proven to be formally exact,^7^ practical realizations rely on an approximation of the unknown exchange correlation (XC) functional 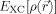 of the charge density 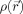 . The functionals most commonly employed in materials science are local (like the local density approximation—LDA^8^ ^9^ ^10^) or semilocal (such as the family of generalized gradient approximations—GGA^11^,^12^), because they combine high numerical performance with often surprisingly good accuracy. In contrast to Hartree–Fock-based approaches, which can be systematically improved by expanding the many-particle wavefunction, a systematic improvement of the XC-functional, although formally possible,^13^ is numerically impractical. A ‘gold standard’ against which the performance of the various functionals can be tested, is therefore not available. As a consequence, it is of paramount importance to carefully check the accuracy and predictive power of the various XC-functionals taking, e.g., selected experimental data into account.

## SINGLE-CRYSTALLINE BULK

Historically, one of the first topics in materials science to which DFT was successfully applied is the phase and lattice stability of ideal crystals, that is, crystals without any defects.^14^ For a material with a given crystallographic structure [e.g., face-centered cubic (fcc), body-centered cubic (bcc), or zincblende (zb)] and in the absence of chemical disorder, the atomic structure can be described by a single parameter—the volume per atom.

**Figure 1:**
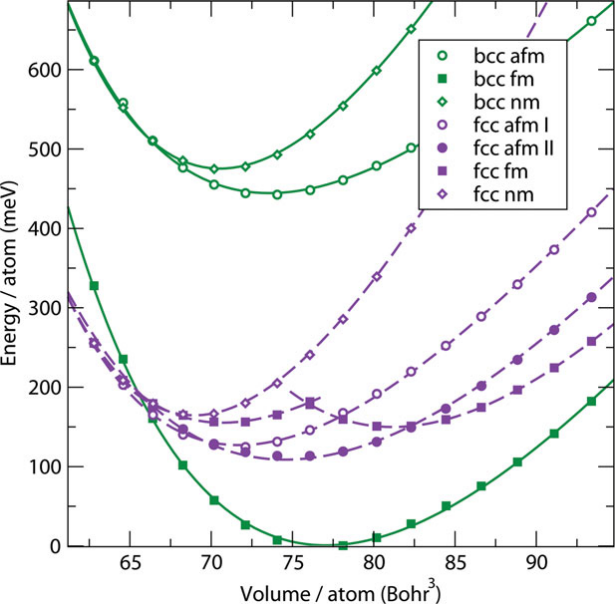
Total energy versus volume curve for two crystallographic (fcc, bcc) and three magnetic structures (non- (nm), ferro- (fm), antiferro- (afm, afmd) magnetic) of single crystalline iron. The calculations provide the equilibrium volume (minimum) of the individual phases as well as information on the crystallographic and magnetic preferences. The example shown here reveals that the *T* = 0 K thermodynamic ground state of bulk iron is the ferromagnetic bcc structure.

Calculating the total energy versus this volume *E*_tot_(*V*) provides important information (see, e.g., Figure 1): The *V* at which *E*_tot_ becomes minimal is the equilibrium volume, *V*_0_, of the corresponding phase. Fitting this curve to the Murnaghan equation of state


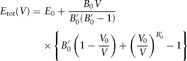
(1)

yields in addition the equilibrium bulk modulus *B*_0_ and its first derivative *B*′_0_. These parameters give the mechanical response of the crystal under hydrostatic load and are important engineering quantities. Considering more complex deformations, the full elastic tensor can be derived. Furthermore, the difference between the minimal total bulk energy *E*_0_ and the atomic energy gives the cohesive energy, an important measure of the chemical bond strength in the crystal. As periodic boundary conditions are used, the actual computational volume is only the (primitive) unit cell that consists of one (e.g., for fcc) or two (e.g., for zb) atoms making such calculations numerically very efficient.

**Figure 2:**
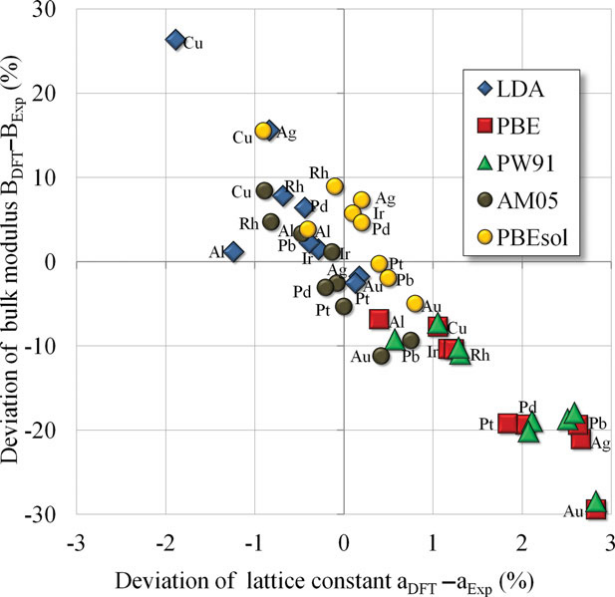
Relative errors between density functional theory computed and experimental bulk moduli (*y*-axis) and lattice constants (*x*-axis). Local density approximation and various generalized gradient approximations for the exchange correlation functional have been employed (PBE,^12^ PW91,^15^ AM05,^16^ PBEsol^17^). The figure is adapted from Refs 18 and 19.

Figure 2 compares data for bulk modulus, *B*, and equilibrium lattice constants, *a*_lat_, calculated using various XC-functionals with experiment. The errors shown are characteristic for these quantities: lattice constants, bond lengths, and so forth can be determined with an accuracy of better than a few percent. In contrast, elastic properties are more sensitive, resulting in errors of ±30%. The trends shown in Figure 2 for the various functionals are well understood and related to specific deficiencies of the respective functionals. LDA tends to yield an overbinding, which results in too strong chemical bonds, too short lattice constants/bond lengths, and, consequently, too high (stiff) bulk moduli. In contrast, GGA is known to lead generally to underbinding and thus to too soft bulk moduli. It is important to note that there are prominent exceptions of these trends, such as Fe, which even in GGA is predicted to have a too small lattice constant.

## CHEMICAL ALLOYS

Most of the metallic materials (such as, e.g., steels) exist as solid solutions of various alloying components, rather than in a stoichiometric phase. Consequently, the distribution of the chemical species over the available lattice sites is fully or partially disordered. Disorder is further relevant for magnetic materials in a paramagnetic state, where the magnetic moments of the individual atoms point in random directions. For both aspects, a few methods that can be combined with DFT have been established in the last decades.

### Coherent Potential Approximation

In the coherent potential approximation (CPA), the concept of an effective medium is used, where the lattice sites are indistinguishable and represented by a mixture of the ordered alloy components. The corresponding coherent potential is self-consistently determined from DFT energies such that the interaction of electrons with individual atoms averages to zero.^20^ The CPA approach is most easily implemented in DFT codes that are based on a Green's function formulation.^21^,^22^ Examples are the Korringa–Kohn–Rostoker (KKR) technique and the linear muffin-tin orbital (LMTO) method.^23^,^24^ More recently, the application of CPA has been extended to exact muffin-tin orbital (EMTO) basis sets,^25^ which allows all-electron precision and, therefore, even the treatment of anisotropic lattice distortions. A major advantage of CPA is its high numerical efficiency. A disadvantage is the conceptional difficulty in including deviations due to local atomic relaxations (as occurring in disordered alloys) from the ideal lattice structure.

Typical materials science problems to which CPA in combination with DFT is applied are provided in Table 2.

**Table 2:** Fields in Materials Science to Which the Coherent Potential Approximation can be Applied

Category	Applications	Material Systems
Structural	Lattice constants	Substitutionally disordered bulk materials
Thermodynamic	Mixing enthalpies	Substitutionally disordered bulk materials
Electronic	Width of bandgap	Off-stoichiometric ternary semiconductors
Mechanical	Elastic constants	Intermetallic compounds
Dimensional	Surface energies	Substitutionally disordered bulk materials

### Special Quasirandom Structures

Chemical disorder can also be modeled, if the DFT calculations are performed with sufficiently large supercells. The degree of artificial order in a (small, periodically repeated) supercell is quantified by correlation functions attributed to a selected set of structural motives. The atomic configurations for which these values are closest to an infinite random alloy are called special quasirandom structures (SQS).^26^

### Cluster Expansion

The results of DFT calculations for various atomic configurations can be effectively generalized, if these configurations are decomposed into structural motives within a cluster expansion (CE). In this approach, an Ising-like Hamiltonian is used to parameterize the total energy of a system^27^,^28^:


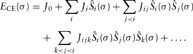
(2)

For the special case of a binary alloy A_x_B_y_, the pseudo spin variables 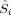 of a particular configuration *σ* are +1 or −1, if site *i* is occupied by atom A or B, respectively. The mapping (2) is exact, if the number of interaction energies *J* is identical to the number of atomic configurations. The idea of the method is, however, to truncate the expansion to a limited set of relevant motives. In this case, a fitting of the *J* values to a relatively small number of direct DFT calculations provides an efficient way to determine the energy of a large set of atomic configurations. The predictive power of this expansion is typically tested by cross-validation schemes. It has been shown that in many cases reliable results require the consideration of concentration-dependent interaction energies.^29^ Besides ground-state formation energies, the CE has, for example, also been used to determine the configurational entropy of alloys, configuration-dependent elastic properties, or the energetics of spin configurations.

## POINT DEFECTS

Point defects are commonly classified into native defects (vacancies, interstitials, antisites), if they are lattice defects involving no external impurities, and extrinsic defects, if impurities are substitutionally or interstitially incorporated. Key quantities characterizing point defects are their concentration, their electrical activity in semiconductors and ceramics, and their induced volume changes.

### Fundamentals

To determine the above quantities, we need to compute the defect formation energy^30^:


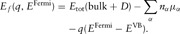
(3)

**Figure 3:**
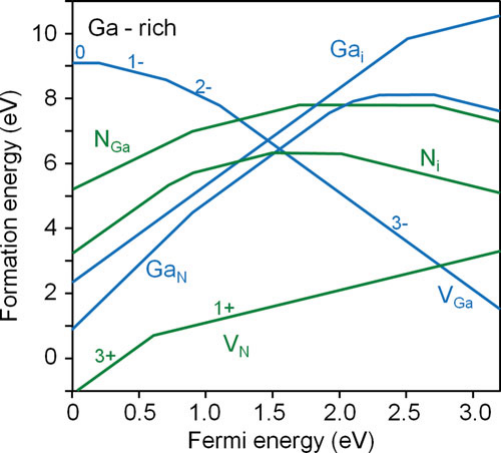
Formation energy of various point defects in bulk GaN as function of the Fermi energy 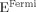 . V_N_ and V_Ga_ are N and Ga vacancies, N_Ga_ and Ga_N_ antisites (a N(Ga) atom on a Ga(N) site respectively), and N_i_ and Ga_i_ the corresponding interstitials. The numbers give the (energetically) most favorable charge state of the respective defect. The kinks in the formation energies give the position of the electronic charge transfer level. (Reproduced with permission from Ref 30. Copyright 2004, American Institue of Physics.)

Here, *q* is the charge state of the defect (for metals, *q* = 0), *E*_tot_(bulk + *D*) is the total energy of the bulk system with defect *D*, 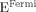 and 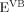 are the Fermi energy and the energy at the top of the valance band respectively, and *n_α_* and *μ_α_* are the number of atoms in the supercell and the chemical potential of species α, respectively. For conditions at which the defects are in thermodynamic equilibrium with the environment (i.e., high temperatures, long timescales), exact boundary conditions and relations between the chemical potentials can be formulated. To be more specific, an ordered binary compound AB with impurity D is considered: ensuring thermodynamic stability of the AB compound with the chemical potentials of its constituents enforces μ_AB_ = μ_*A*_ + μ_*B*_ with μ_AB_ the chemical potential of the bulk compound. To prevent decomposition of the system into a mixture of the bulk compound and a pure A or B phase an upper limit for any potential is μ_A_ < μ_A (bulk)_ with μ_α(bulk)_ being the chemical potential of the bulk (or gas) phase of the respective constituent. Finally, to avoid formation of undesired precipitate phases the chemical potentials have to obey different bounds, for example, 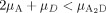 to prevent the formation of an A_2_D phase. Using this approach, realistic environments can be modeled in a transparent way. For an example, the formation energies of point defects in GaN are provided in Figure 3.

Knowing the defect formation energy, the equilibrium defect concentration can be directly determined:


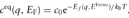
(4)

Here, *c*_0_ is the concentration of possible sites where the defect can be formed, *k*_B_ is the Boltzmann constant, and *T* is the temperature. For metals, the dependence on the Fermi energy 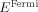 (which is the energetic position of the electron reservoir) and thus on the charge state *q* drops: The equilibrium concentrations of the defects are thus directly determined. For semiconductors/ceramics, the Fermi level can vary between the top of the valence band (highest occupied molecular orbital, HOMO) and the lowest unoccupied state (lowest unoccupied molecular orbital, LUMO). The position of the Fermi level is given by the charge neutrality condition, that is, the sum over all charges in the system (defects, electrons in the conduction band, holes in the valence band) must be zero.^30^ In addition, the Fermi level where the formation energy of two charge states *q*, *q*′ of the same defect equals is the charge transfer level *E*^*q*, *q*^′. Knowing the position of all charge transfer levels allows one to determine the electronic behavior of the defect, that is, whether the defect behaves as donor, acceptor, or amphoteric defect that both accepts and donates electrons.

### Supercell Convergence

The most common approach to compute the above defined defect formation energies is the supercell approach. In this approach, a large bulk cell consisting of *O*(10^2^) atoms and with a single defect in it is periodically repeated. Because the defect concentration in such a cell is typically orders of magnitude larger than realistic defect concentrations, the interaction of the defect with its periodic images is spurious and must be corrected. The interaction of the defect with its images occurs via three mechanisms.: first, electronic interactions due to the overlap of the defect wave functions. The importance of this effect can be seen by inspecting the dispersion of the defect band. Second, elastic interactions induced by atomic relaxations around the defect. Third, if the defect is charged there is a long-range Coulomb interaction with the neighboring defects and the compensating background. For all three interactions, efficient correction schemes have been proposed that allow the use of modest supercell sizes in determining the correct formation energy of an isolated defect in an infinitely large supercell.^131^ ^32^ ^33^

### Overcoming the Bandgap Problem

A major hurdle in computing formation energies and charge transfer levels of defects in semiconductors is the infamous bandgap problem in DFT when using semilocal XC-functionals such as LDA or GGA. Because errors in the theoretical bandgap can be in the range of several eV, similar errors are expected in the computed formation energies. Recently, thanks to hugely improved CPU power and algorithmic developments, DFT approaches with hybrid XC-functionals (mixing exact exchange and semilocal XC-functionals)^34^,^35^ and, to a lesser extent, quasiparticle (GW)^36^ and quantum Monte Carlo (QMC)^37^,^38^ calculations can be used to compute the large supercells needed to describe point defects. These approaches largely overcome the bandgap problem (typical errors are <0.1 eV), correctly describe localization phenomena at single dangling bond states, and are generally believed to accurately describe defect energetics.

## EXTENDED DEFECTS

Extended defects are commonly categorized into one-dimensional line defects (dislocations) and two-dimensional defects such as defects in the crystallographic structure (stacking faults), grain boundaries (occurring between two crystallites of the same phase, e.g., in polycrystals), interfaces (boundary between two different phases), and internal and external surfaces and quasi-zero-dimensional defects (precipitates, quantum dots).

### Dislocations

Dislocations play a key role in understanding plasticity in structural materials^39^ and are also well known to adversely affect electronic and optical properties and lifetime of modern semiconductor devices.^40^

To understand plasticity a key parameters that needs to be modeled is the mobility (activation energy) of dislocations under applied strain. Because the mobility is strongly affected by the nucleation of dislocation kinks, interactions with impurities/precipitates, and phonon drag (frictional forces due to the interaction with phonons), large system sizes (>10^3^…10^4^ atoms) and large timescales (∼10 ps) are needed. This makes such calculations impractical for today's DFT approaches and most computations on this topic have been performed using empirical potentials (see, e.g., Ref 41). Only recently with the advent of multiscale techniques could critical aspects of dislocation mobility be successfully addressed using DFT (see, e.g., Ref 42).

To understand the impact of dislocations on electronic/optical properties their atomic geometry and electronic structure need to be determined. For straight dislocations (i.e., in the absence of any kinks), the required atomic relaxation of a few internal dislocation core structures can be accomplished using DFT. To avoid artifacts due to the slowly decaying 1/*r* (with *r* the distance to the dislocation core) stress field around a dislocation, various schemes have been developed to minimize spurious interactions with periodic images.^43^,^44^

### Stacking Faults, Grain Boundaries, Interfaces, and Surfaces

Ideal planar defects, which are free of kinks or steps, can exploit the periodic boundary conditions common to most DFT codes. For such structures, only the direction normal to the defect shows no periodicity and requires a sufficiently large supercell dimension to avoid image artifacts. In practice, 5–10 atomic layers are often sufficient to separate interactions between the extended defects in neighboring supercells.^45^ For high-symmetry static planar defects such as, for example, stacking faults or unreconstructed surfaces supercells as small as *O*(10) atoms are sufficient to obtain converged results with respect to equilibrium atomic geometry, electronic structure and energy. These quantities are of high relevance in addressing, for example, the properties of semiconductor heterointerfaces (valence and conduction band offset, impurity segregation), catalytic activity on surfaces, or mechanical strength of polycrystals or precipitate strengthened alloys.

Imperfections on planar defects such as steps and kinks are important in understanding the dynamic behavior of such defects but can also strongly affect the local chemical activity. Including such structural features requires large supercell sizes of more than a few hundred atoms, which can be still handled by modern DFT approaches. Modeling the time evolution is critical in understanding, for example, epitaxial surface growth (which is the preferred fabrication technique to make optoelectronic devices) or grain coarsening which critically affects microstructure and thus mechanical stability of structural materials. Relevant timescales can be as slow as a fraction of an atomic layer per second (e.g., semiconductor surface growth) requiring advanced multiscale approaches that couple, for example, DFT calculated single atom diffusion rates with efficient statistical approaches such as kinetic Monte Carlo.^46^ For the much faster dynamics of grain boundaries in metals commonly empirical potentials are used.^47^

## FREE ENERGIES

The original formulation of DFT was for the *T* = 0 K ground state.^7^ Although it can be shown that the concept can be extended to finite electronic temperatures,^48^ historically most calculations were restricted to *T* = 0 K properties due to computational limitations.

### Fundamentals

The key quantity for computing finite temperature properties is the partition function:


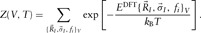
(5)

Here, 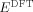 is the DFT computed total energy of a snapshot configuration with atoms *I* at positions 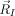 and with magnetic spins 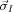, *f_i_* is the occupation number of the electronic state *i*, *V* is the volume of the specific configuration, and *T* the temperature. Knowing the partition function provides direct access to the free energy and other finite temperature properties. Because the dynamics of the electrons is much faster than that of the atoms/magnetic moments (i.e., applying the Born–Oppenheimer approximation) each configuration can be computed in the ground state or the equilibrated finite temperature state using conventional DFT.

A major challenge in computing free energies based on the above formulation is the huge number of configurations needed to achieve statistically converged results. A major reduction is achieved by assuming an adiabatic decoupling of the various excitation channels such as electronic (*f_i_*), magnetic 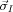 ), or vibrational 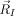 ) excitations. In addition, for point defects and alloys (see corresponding sections above) where many geometrically equivalent configurations exist configurational entropy has to be included. The free energy can then be written as 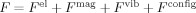 where the triple sum in Eq. (5) reduces to three single sums.

### Electronic Excitations

The electronic free energy is given by


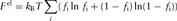
(6)

with the occupation numbers *f*_*i*_ given by the Fermi–Dirac distribution. This contribution is included in most DFT codes and computationally inexpensive.

### Vibronic Excitations

In DFT, the vibronic free energy is commonly computed by expanding the total energy around the equilibrium configuration in a Taylor series of small displacements. Because the first-order contribution vanishes by definition for an equilibrium configuration (which is a local minimum), the dominant term is the second-order contribution (the harmonic contribution). Neglecting higher-order contributions, the harmonic free energy can be written as


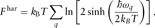
(7)

where ω_*q*_ are the phonon energies obtained from diagonalizing the dynamical matrix and 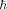 is Planck's constant. A large part of the anharmonic contributions can be included by computing the volume dependence of Eq. (7), that is, by computing how the phonon energies change when the volume increases due to thermal expansion. This approximation is called the quasiharmonic approximation and allows the computation of temperature-dependent materials properties such as thermal expansion, elastic constants (e.g., bulk modulus), or isobaric/isochoric heat capacities. The quasiharmonic approximation requires as its only input the phonon frequencies for a few (5–10) volumes. Two major techniques are used to compute phonon spectra—linear response techniques where the calculations can be performed in the elementary unit cell^49^ and direct approaches where the response of the system onto a small perturbation is computed in a large supercell.^50^ The advantage of the first formalism is that for simple bulk cells often only a single-atom cell is needed, whereas the advantage of the second approach is that any DFT code providing forces can be used. For bulk crystals, both techniques are nowadays quite affordable.

For high temperatures above ∼0.7…0.8 *T* 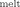, explicit anharmonic contributions become relevant. Estimates show that to compute derivatives of the free energy such as heat capacities, statistical errors in the free energy should be well below 1 meV.^51^ To achieve such an accuracy, direct configuration space sampling such as Eq. (5) would require 10^6^…10^7^ configurations making it unfeasible with a direct DFT approach. Modern sophisticated sampling techniques allow the targeted accuracy to be achieved with a few hundred carefully constructed configurations.^52^

### Magnetic Excitations

Magnetic phenomena can be classified into diamagnetism, paramagnetism, and collective magnetic ordering, such as ferromagnetism, ferrimagnetism, or antiferromagnetism. Furthermore, one needs to distinguish between materials with localized magnetic moments (caused by partially filled inner electron shells) and itinerant moments (carried by conduction electrons). The dominant magnetic excitations in systems with a collective magnetic ordering are (collective) spin-wave excitations or (single-particle) Stoner excitations. In many materials, spin-wave excitations of local moments dominate the thermodynamic properties. Their energetics is most often described using a Heisenberg model


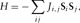
(8)

with spin operators **S**_*i*_ for the localized spins and exchange integrals *J*_*i,j*_, which in the most general case also depend on the value of the localized magnetic moments. Different DFT methods are available to determine the exchange integrals *J*_*i,j*_, allowing an *ab initio*-based description of magnetic excitations. The most prominent ones are the application of the magnetic force theorem,^53^ the application of KKR methods or the calculation of frozen spin waves.

The spin Hamiltonian Eq. (8) can be solved by applying analytical (such as the random phase approximation^54^) or numerical (such as classical or quantum Monte Carlo^55^) methods. Recent studies show that spin quantization effects (the quantum character of the spin operators **S**_*i*_) cannot be neglected when computing free energies, heat capacities or magnetization curves below the Curie temperature.^56^

### Other Free Energy Contributions

Beyond the three typically dominant excitation mechanisms discussed above several additional mechanisms exist in realistic materials. Due to their large configurational entropy point defects can occur at high concentrations (10^−3^…10^−4^) at temperatures close to the melting temperature and may impact thermodynamic properties such as heat capacities. In contrast, the configurational entropy of extended defects is too small for them to exist in thermodynamic equilibrium.

Although the adiabatic decoupling of vibrational, magnetic, and electronic degrees is very popular due to its often high predictive power (see below) and its computational efficiency, materials and conditions exist where nonadiabatic couplings between these excitations cannot be neglected. Examples are super­conductors (electron–phonon coupling) and multiferroics or magnetic structural materials such as steels (magnon–electron–phonon coupling).

### Accuracy of Finite Temperature Calculations

Recent methodological and computational advances provided the opportunity to compute fully converged (including statistical averages) free energies and derived materials properties, such as heat capacities.^18^ For such calculations, the only unavoidable error that remains is due to the choice of the DFT XC-functional. Comparing with high-quality experimental data thus provides an efficient route to assess the quality of various XC-functionals, which all have been constructed for *T* = 0 K, in predicting thermodynamic quantities.

Recent studies^18^ indicate that the magnitude of the deviation between LDA and various GGA functionals in the temperature dependence of various bulk properties (heat capacity, free energy, thermal expansion) provides an approximate indicator for DFT error bars. These studies also show that GGA does not outperform LDA for finite temperature calculations. Presently, it is therefore highly advisable to perform such calculations with at least two XC-functionals and to carefully monitor deviations.

**Figure 4:**
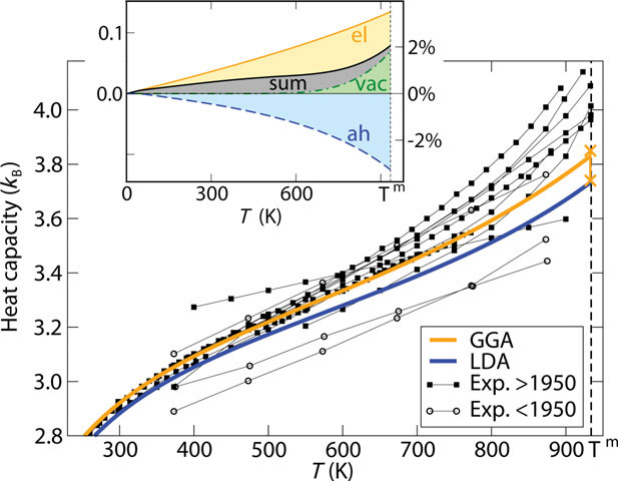
Isobaric heat capacity of aluminum including the quasiharmonic, electronic, anharmonic, and vacancy contributions compared to experiment. The modification due to the last three contributions with respect to the quasiharmonic result is for the case of the generalized gradient approximations shown in the inset (note the different scale). The melting temperature *T*^m^ of Al (933 K) is indicated by a vertical dashed line. (Adapted with permission from Ref 59. Copyright 2011, IOP publishing. References for the experimental data can be found in Ref 57.)

**Figure 5:**
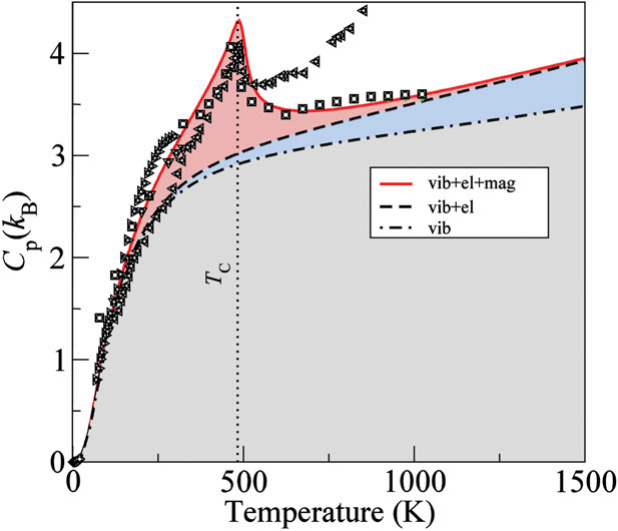
Calculated heat capacity (lines) of cementite in comparison with available experimental data (open symbols). The calculated vibrational, electronic, and magnetic contributions to the heat capacity are shown in shaded gray (lower area), blue (middle), and red (upper area) correspondingly. (Adapted with permission from Ref 59. Copyright 2011, IOP publishing. References for the experimental data can be found in Ref 58.)

The presently achievable accuracy of common XC-functionals is exemplary shown in Figure 4 for the experimentally relevant isobaric heat capacity of aluminum: The scatter in the experimental data is substantially larger than the estimated DFT error bar (difference between the LDA and GGA functional). The accuracy achievable with these DFT techniques opens new opportunities to identify the mechanisms driving the instability of materials at high temperatures^52^ or can even be used to assess the quality of experimental thermodynamic data (see Figure 5). As all free energy contributions are included, they also provide a unique insight into the relative importance and temperature dependence of the various contributions (see Figure 5).

## CONCLUSIONS

DFT has emerged as a powerful technique in materials science for addressing a wide range of topics. Recent years have seen an enormous progress in overcoming early challenges such as the infamous bandgap problem for semiconductors or the restriction to *T* = 0 K ground-state calculations when considering realistic materials. Still, many challenges are left for future research such as the development of improved XC-functionals for predicting phase transition temperatures with an accuracy of ∼20 K (as, e.g., needed for technical thermodynamic databases), the efficient sampling of huge chemical and structural configuration spaces (as crucial for technical materials), or going to realistic length and timescales by developing efficient multiscale strategies.

## References

[1] Kresse G, Furthmüller J (1996). Efficiency of ab-initio total energy calculations for metals and semiconductors using a plane-wave basis set. Comput Mater Sci.

[2] Gonze X, Beuken JM, Caracas R, Detraux F, Fuchs M, Rignanese GM, Sindic L, Verstraete M, Zerah G, Jollet F (2002). First-principles computation of material properties: the ABINIT software project. Comput Mater Sci.

[3] Blaha P, Schwarz K, Sorantin P, Trickey SB (1990). Full-potential, linearized augmented plane-wave programs for crystalline systems. Comput Phys Commun.

[4] Artacho E, Anglada E, Diéguez O, Gale JD, Garcia A, Junquera J, Martin RM, Ordejón P, Pruneda JM, Sánchez-Portal D (2008). The SIESTA method; developments and applicability. J Phys: Condens Matter.

[5] Bowler DR, Fattebert JL, Gillan MJ, Haynes PD, Skylaris CK (2008). Introductory remarks: linear scaling methods - Preface. J Phys: Condens Matter.

[6] Bowler DR, Miyazaki T (2012). O(*N*) methods in electronic structure calculations. Rep Prog Phys.

[7] Hohenberg P, Kohn W (1964). Inhomogeneous electron gas. Phys Rev.

[8] Kohn W, Sham LJ (1965). Self-consistent equations including exchange and correlation effects. Phys Rev.

[9] Ceperley DM, Alder BJ (1980). Ground-state of the electron-gas by a stochastic method. Phys Rev Lett.

[10] Perdew JP, Zunger A (1981). Self-interaction correction to density-functional approximations for many-electron systems. Phys Rev B.

[11] Perdew JP, Chevary JA, Vosko SH, Jackson KA, Pederson MR, Singh DJ, Fiolhais C (1992). Atoms, molecules, solids, and surfaces—applications of the generalized gradient approximation for exchange and correlation. Phys Rev B.

[12] Perdew JP, Burke K, Ernzerhof M (1996). Generalized gradient approximation made simple. Phys Rev Lett.

[13] Gorling A, Levy M (1994). Exact Kohn–Sham scheme based on perturbation theory. Phys Rev A.

[14] Yin MT, Cohen ML (1980). Microscopic theory of the phase-transformation and lattice-dynamics of SI. Phys Rev Lett.

[15] Perdew JP, Wang Y (1992). Pair-distribution function and its coupling-constant average for the spin-polarized electron-gas. Phys Rev B.

[16] Armiento R, Mattsson AE (2005). Functional designed to include surface effects in self-consistent density functional theory. Phys Rev B.

[17] Perdew JP, Ruzsinszky A, Csonka GI, Vydrov OA, Scuseria GE, Constantin LA, Zhou XL, Burke K (2008). Restoring the density-gradient expansion for exchange in solids and surfaces. Phys Rev Lett.

[18] Grabowski B, Hickel T, Neugebauer J (2007). Ab initio study of the thermodynamic properties of nonmagnetic elementary fcc metals: exchange-correlation-related error bars and chemical trends. Phys Rev B.

[19] Nazarov R, Hickel T, Neugebauer J (2012). Vacancy formation energies in fcc metals: influence of exchange-correlation functionals and correction schemes. Phys. Rev. B.

[20] Soven P (1967). Coherent-Potential model of substitutional disordered alloys. Phys Rev.

[21] Gunnarsson O, Jepsen O, Andersen OK (1983). Self-consistent impurity calculations in the atomic-spheres approximation. Phys Rev B.

[22] Abrikosov IA, Skriver HL (1993). Self-consistent linear-muffin-tin-orbitals coherent-potential technique for bulk and surface calculations—Cu-Ni, Ag-Pd, and Au-Pt random alloys. Phys Rev B.

[23] Faulkner JS, Stocks GM (1980). Calculating properties with the coherent-potential approximation. Phys Rev B.

[24] Skriver HL (1984). The LMTO Method: Muffin-Tin Orbitals and Electronic Structure.

[25] Vitos L (2001). Total-energy method based on the exact muffin-tin orbitals theory. Phys Rev B.

[26] Zunger A, Wei SH, Ferreira LG, Bernard JE (1990). Special quasirandom structures. Phys Rev Lett.

[27] Connolly JWD, Williams AR (1983). Density-functional theory applied to phase transformations in transition-metal alloys. Phys Rev B.

[28] Sanchez JM, Ducastelle F, Gratias D (1984). Generalized cluster description of multicomponent systems. Physica A.

[29] Sanchez JM (2010). Cluster expansion and the configurational theory of alloys. Phys Rev B.

[30] Van de Walle CG, Neugebauer J (2004). First-principles calculations for defects and impurities: applications to III-nitrides. J Appl Phys.

[31] Makov G, Payne MC (1995). Periodic boundary-conditions in ab-initio calculations. Phys Rev B.

[32] Lany S, Zunger A (2008). Assessment of correction methods for the band-gap problem and for finite-size effects in supercell defect calculations: case studies for ZnO and GaAs. Phys Rev B.

[33] Freysoldt C, Neugebauer J, Van de Walle CG (2009). Fully ab initio finite-size corrections for charged-defect supercell calculations. Phys Rev Lett.

[34] Heyd J, Scuseria GE, Ernzerhof M (2003). Hybrid functionals based on a screened Coulomb potential. J Chem Phys.

[35] Oba F, Togo A, Tanaka I, Paier J, Kresse G (2008). Defect energetics in ZnO: a hybrid Hartree–Fock density functional study. Phys Rev B.

[36] Rinke P, Janotti A, Scheffler M, Van de Walle CG (2009). Defect formation energies without the band-gap problem: combining density-functional theory and the GW approach for the silicon self-interstitial. Phys Rev Lett.

[37] Leung WK, Needs RJ, Rajagopal G, Itoh S, Ihara S (1999). Calculations of silicon self-interstitial defects. Phys Rev Lett.

[38] Batista ER, Heyd J, Hennig RG, Uberuaga BP, Martin RL, Scuseria GE, Umrigar CJ, Wilkins JW (2006). Comparison of screened hybrid density functional theory to diffusion Monte Carlo in calculations of total energies of silicon phases and defects. Phys Rev B.

[39] Vitek V (1992). Structure of dislocation cores in metallic materials and its impact on their plastic behaviour. Prog Mater Sci.

[40] Hirth JP, Lothe J (1982). Theory of Dislocations.

[41] Bulatov VV, Yip S, Argon AS (1995). Atomic modes of dislocation mobility in silicon. Philos Mag A.

[42] Warner DH, Curtin WA, Qu S (2007). Rate dependence of crack-tip processes predicts twinning trends in f.c.c. metals. Nat Mater.

[43] Lymperakis L, Neugebauer J, Albrecht M, Remmele T, Strunk HP (2004). Strain induced deep electronic states around threading dislocations in GaN. Phys Rev Lett.

[44] Trinkle DR (2008). Lattice Green function for extended defect calculations: computation and error estimation with long-range forces. Phys Rev B.

[45] Lymperakis L, Abu-Farsakh H, Marquardt O, Hickel T, Neugebauer J (2011). Theoretical modeling of growth processes, extended defects, and electronic properties of III-nitride semiconductor nanostructures. Phys Status Solid B.

[46] Kratzer P, Scheffler M (2002). Reaction-limited island nucleation in molecular beam epitaxy of compound semiconductors. Phys Rev Lett.

[47] Gottstein G, Shvindlerman LS (1999). Grain Boundary Migration in Metals: Thermodynamics, Kinetics, Applications.

[48] Mermin ND (1965). Thermal properties of inhomogeneous electron gas. Phys Rev.

[49] Giannozzi P, Degironcoli S, Pavone P, Baroni S (1991). Ab initio calculation of phonon dispersions in semiconductors. Phys Rev B.

[50] Kresse G, Furthmüller J, Hafner J (1995). Ab initio force constant approach to phonon dispersion relations of diamond and graphite. Europhys Lett.

[51] Grabowski B, Söderlind P, Hickel T, Neugebauer J (2011). Temperature-driven phase transitions from first principles including all relevant excitations: the fcc-to-bcc transition in Ca. Phys Rev B.

[52] Grabowski B, Ismer L, Hickel T, Neugebauer J (2009). Ab initio up to the melting point: anharmonicity and vacancies in aluminum. Phys Rev B.

[53] Liechtenstein AI, Katsnelson MI, Gubanov VA (1984). Exchange Interactions and Spin-Wave Stiffness in Ferromagnetic Metals. J Phys F Metal Phys.

[54] Körmann F, Dick A, Grabowski B, Hallstedt B, Hickel T, Neugebauer J (2008). Free energy of bcc iron: Integrated ab initio derivation of vibrational, electronic, and magnetic contributions. Phys Rev B.

[55] Alet F, Lucini B, Vettorazzo M (2005). A cluster algorithm for lattice gauge theories. Comput Phys Commun.

[56] Körmann F, Dick A, Hickel T, Neugebauer J (2011). Role of spin quantization in determining the thermodynamic properties of magnetic transition metals. Phys Rev B.

[57] Grabowski B, Hickel T, Neugebauer J (2011). Formation energies of point defects at finite temperatures. Phys Status Solidi B.

[58] Dick A, Körmann F, Hickel T, Neugebauer J (2011). Ab initio based determination of thermodynamic properties of cementite including vibronic, magnetic, and electronic excitations. Phys Rev B.

[59] Hickel T, Grabowski B, Körmann F, Neugebauer J (2012). Advancing density functional theory to finite temperatures: methods and applications in steel design. J Phys: Condens Matter.

